# Deus Sine Machina: Which Intelligence, for Whom

**DOI:** 10.1111/den.70265

**Published:** 2026-08-23

**Authors:** Eisuke Iwasaki, Aya Kawanishi, Fateh Bazerbachi

**Affiliations:** ^1^ Division of Gastroenterology and Hepatology, Department of Internal Medicine Tokai University School of Medicine Isehara Japan; ^2^ CentraCare, Interventional Endoscopy Program St. Cloud Hospital St. Cloud Minnesota USA; ^3^ Division of Gastroenterology, Hepatology and Nutrition University of Minnesota Minneapolis Minnesota USA

Artificial intelligence (AI) has advanced rapidly in gastrointestinal endoscopy. In colonoscopy, computer‐aided detection raises adenoma detection across many trials [1], and AI is extending to lesion characterization, quality control, documentation, and education [2]. In this issue of *Digestive Endoscopy*, Coelho‐Prabhu et al. present the World Endoscopy Organization's (WEO) response to the World Health Organization's Global Initiative on AI for Health, a welcome insistence that AI be judged from the standpoint of global equity rather than the frontier of the high‐resource world alone [3]. The WEO is right that, even as AI must escape the preserve of the affluent world, its naïve export may widen rather than narrow disparities. We carry the argument one step further, toward the question the statement raises but leaves implicit: not whether AI can be made to work in low‐ and middle‐income countries, but which AI, for which disease, and for which unmet need.

That AI tends to refine advanced systems rather than reach those most in need is not peculiar to AI; it is the latest form of a structural feature of biomedical innovation, the 10/90 gap, in which scarcely a tenth of research spending is directed at the diseases responsible for the great majority of the world's suffering. AI threatens not merely to inherit this gap but to widen it, for its returns accrue where data and infrastructure already exist, concentrating its gains where capability is already greatest.

It helps to distinguish two margins on which AI can operate. In the high‐resource setting it works on the intensive margin, refining a capability that already exists, such as lifting adenoma detection from good to better. In much of the world the relevant margin is extensive: the capability does not exist at all, and to deploy there is not to refine a service but to create one. An algorithm that improves adenoma detection where no colonoscopy exists refines a service the population has never had; the prior task is not refinement but provision, of a basic and lifesaving capability that is wholly absent. The mismatch is twofold, for endoscopic AI has been built largely around the conditions that dominate high‐income practice, colorectal cancer, Barrett's esophagus, inflammatory bowel disease, rather than the esophageal and gastric cancers and the hepatitis‐B and schistosomal cirrhosis that carry the heaviest burden where resources are thinnest.

Therefore, our most refined tools, ported identically, would be misplaced twice over: the wrong margin, and the wrong disease.

The point is concrete. Take endoscopic retrograde cholangiopancreatography (ERCP) for ascending cholangitis: it addresses a lethal condition, yet across much of sub‐Saharan Africa this capability is scarce or absent. In a survey of physicians across four eastern African countries, of 87 who responded, 63 performed endoscopy, but only six performed ERCP and two performed endoscopic ultrasound (EUS) [4]. Where programs exist they have been built deliberately and against the grain: at Tenwek Hospital in rural Kenya, a sustained ERCP service was established over nearly a decade, 277 procedures whose commonest indication was malignancy or stones [5]. Such programs remain the exception, and the extension of ERCP into countries such as Mozambique is only now beginning [6]. For a patient with cholangitis where no one is trained to cannulate, the limiting reagent is the operator, and the most refined diagnostic classifier does nothing for her.

This reframes what AI should be asked to do where resources are thin: could AI furnish an absent capability, rather than merely refine an existing one? We believe it can, and propose four potential domains. The first attacks the operator constraint directly: a proctor at the elbow where training is scarce, AI guides the hand through the procedure itself and shortens the curve until local operators gain independence. The template exists: deep‐learning systems that distinguish malignant from benign biliary strictures [7], and EUS‐based models to assist with gallbladder drainage [8]. These invert the high‐income paradigm: there such tools refine an already‐supervised trainee, while where expertise is scarce the same guidance, cannulation assistance, vessel‐avoidance overlays, station recognition, may decide whether a procedure is offered at all. The second is pathology substitution: where pathologists are scarcer than endoscopists and tissue turnaround is measured in weeks, AI optical diagnosis that lets a confidently low‐risk lesion be resected and discarded does not merely economize; it makes a cancer screening program viable where none could otherwise exist. The third is triage, where endoscopy itself is scarce: AI on cheap, ubiquitous inputs, laboratory values, ultrasound, elastography, can ration the one available scope, directing variceal screening in cirrhosis to the patients who need treatment of high risk lesions. The fourth extends detection beyond the endoscopy suite, applying AI to smartphone images, disposable capsule, or transoral brushings in ambulatory clinics, and referring upward only those who screen positive, which matters most for the cancers that define the regional burden, such as the esophageal squamous‐cell carcinoma of the East African corridor [9]. These roles are complementary rather than exclusive, screening feeding triage rather than standing apart from it, and they differ in maturity: the first two already rest on emerging clinical evidence, the latter two remain largely conceptual and await real‐world validation.

This promise carries its own hazard, which the WEO statement rightly flags [3]. The tool that lowers the threshold for a less experienced operator can, when inaccurate, mislead and a confident error has nowhere to be caught. Overreliance, deskilling, and a distorted learning curve, buffered in well‐resourced units by supervision, second readers, and fallback safeguards, strike hardest where those buffers are absent, so a flawed model does not merely miss a lesion but teaches the wrong technique to a trainee who has no other teacher. The corollary is a higher bar, not a lower one: AI meant to build operators where there are none must meet a stricter standard than the same tool would face assisting an expert.

Each proposal meets a natural limit: AI is software. It cannot manufacture a duodenoscope, a fluoroscopy unit, or a stent; the gallbladder‐drainage assistant presupposes the very lumen‐apposing stent it deploys, which no algorithm will conjure. Beneath every intervention lies a material substrate AI does not cross.

Yet AI acts on two of the hardware problem's three faces. The first is substitution: a cheaper, more available modality can stand in for an absent expensive one. EUS detects biliary stones and pancreatic lesions where magnetic resonance imaging is unavailable, and the examination that looks for hepatic malignancy can screen the same patient for varices, one instrument answering two questions. Its weakness, operator dependence, is precisely the variable AI reduces. The second face is conservation: where every consumable is precious, choosing the right device is itself an economy. How many stents a hilar stricture needs and where they should sit; what will pass a tight stenosis and to what length it should be cut; what will span a duodenal or colonic one. What an expert reads at a glance, a novice may not, and a stent deployed too short is a stent lost. AI that measures the target and names the device before it is used spends nothing to save one. The third face AI cannot touch: the endoscope, the fluoroscopy suite, the stent itself.

None of this removes the implementation requirements the WEO statement enumerates: local datasets and external validation in the target population, training that conveys AI's failure modes, risk‐proportionate regulation, region‐specific cost‐effectiveness, and patient trust [2, 3, 10]. But these are conditions for deploying a tool correctly; they presuppose the right tool has been chosen. The deeper task, which should organize the WEO's pathway, comes earlier: to decide what AI is for in a given setting by reading the local constraint set rather than importing the objective function of the high‐income world. Low‐ and middle‐income countries are not one place: the shortage that binds, whether of operators, pathologists, equipment, or referral pathways, differs between countries and within them, so AI's role follows the local bottleneck rather than the national income label. Who sets the priority therefore matters as much as the priority itself: it should fall to local clinicians, patients, policymakers, and regional societies to name the unmet need, with the WEO convening and shepherding rather than deciding. Two things it can do at once: map the binding constraint region by region through its training networks, and seat its experienced members on local steering committees to draft the target product profiles that define what a tool must do, and for whom, before it is built. Localization is usually understood as porting, taking a model built in one setting and validating it in another. We mean something different: re‐deriving the question the model is meant to answer.

In classical theater, the deus ex machina descended by the *mechane*, a stage crane that brought a god onto the scene to resolve what human effort could not, a divine gift redeeming an otherwise insoluble predicament. But the gift assumes a crane. AI is offered to global health in the same redemptive register, yet it descends only onto stages already fitted to receive it. *Deus sine machina*, a god without a machine, is the condition of most of the world: the salvation cannot arrive, because the apparatus to usher it in is yet to be built. The WEO and its sister societies cannot meet this by waiting for the crane, nor by summoning a ghost that has no machine. They can meet it only by building differently, and by asking first, before implementation, the prior question: which intelligence, and to what end. Whether AI narrows the gap or widens it will not turn on how intelligent our machines become, but it will be settled by our deliberate answer to an older and harder question: for whom.

## Funding

The authors have nothing to report.

## Conflicts of Interest

The authors declare no conflicts of interest.

## Linked Article

This article is linked with Coelho‐Prabhu et al. (2025). To view this article visit http://doi.org/10.1111/den.70011.

## Data Availability

Data sharing not applicable to this article as no datasets were generated or analysed during the current study.
